# Interventions Targeting Quality of Life for Colorectal Cancer Patients with Fecal Ostomy: A Systematic Review

**DOI:** 10.1016/j.gassur.2026.102454

**Published:** 2026-05-14

**Authors:** Stefanie J Soelling, Atziri Rubio-Chavez, Laura Baird Thurman, Colleen Mcdermott, Lisa Philpotts, Mary Brindle, Ana-Maria Vranceanu, Zara Cooper, Christine S. Ritchie, Christy E. Cauley

**Affiliations:** a Department of Surgery, Brigham and Women’s Hospital, Harvard Medical School, Boston, MA, 02115; b Ariadne Labs, Brigham and Women’s Hospital, Harvard. T.H. School of Public Health, Boston, MA, 02115; c Department of Surgery, Massachusetts General Hospital, Harvard Medical School, Boston, MA, 02114; d Center for Health Outcomes and Interdisciplinary Research, Department of Psychiatry, Massachusetts General Hospital, Boston, MA, 02114.; e Harvard Medical School, Boston, MA, 02115.; f The Center for Surgery and Public Health, Brigham and Women’s Hospital, Boston, MA, 02115.; g Marcus Institute for Aging Research, Boston, MA, 02115.; h Mongan Institute Center for Aging and Serious Illness, Massachusetts General Hospital, Boston, MA, 02114.

## Abstract

**Background:**

Patients undergoing fecal ostomy surgery often struggle to adapt to life, particularly when it is performed for colorectal cancer. Maladaptation to life with an ostomy is associated with psychosocial challenges, impacting daily quality of life. The aim of this systematic review is to identify interventions addressing self-care education and psychosocial needs of colorectal cancer patients living with an ostomy and examine their efficacy and effectiveness.

**Methods:**

A systematic search was conducted in Ovid MEDLINE, PsycInfo, Cochrane Clinical Trials, CINAHL, Embase, and Web of Science on September 10, 2025. Randomized control trials, prospective cohort studies, case studies, and retrospective studies of tested interventions used before or after ostomy surgery with specific focus on colorectal cancer patients. Patient reported outcomes, including quality of life (QoL) are summarized.

**Results:**

Fourteen of the twenty-one studies included reported a positive outcome in QoL for the intervention group. The remaining studies reported mixed results or did not use validated QoL measures. Several studies use additional resources, such as trained nurses. No study reported harm to using the intervention.

**Discussion:**

Resource intensive interventions designed to improve colorectal cancer patient quality of life after ostomy surgery show promising improvements in patient-centered outcomes. The majority of studies show improved quality of life or no harm.

**Conclusions:**

Interventions aimed at improving quality of life of colorectal cancer patients with fecal ostomies are resource intensive. Future work is needed to understand the scalability of these interventions to better support these surgical patients.

**PROSPERO number:**

CRD42022382142

## Introduction:

Surgery requiring a fecal ostomy is commonplace, with approximately 100,000 individuals annually across the United States undergoing a procedure with a fecal ostomy,^1^ with cancer being one of the most common indications. Many patients experience difficulty adapting to life with their ostomy. Difficulty adapting has a negative impact on patients’ quality of life^2–6^ in addition to postoperative complications, such as surgical site infections and dehydration after ileostomy.^7^ Many patients experience skin irritation, leakage or issues with their appliances, odor, or noise from the ostomy.^2,3^ Due to these challenges, many patients report less enjoyment from activities and depression or anxiety following surgery with an ostomy.^2^ About half of the patients surveyed had significant postoperative stress.^3^ Nugent et al. found that 80% of patients with an ostomy reported changes to their lifestyle postoperatively.^4^ Male and female ostomy patients both had significantly worse psychosocial wellbeing, with higher reports of depression and suicidal ideation compared to matched controls without ostomies.^8^

There is a gap in preoperative and postoperative education and psychosocial support to improve patient-centered outcomes after surgery with an ostomy particularly for patients with colorectal cancer. A recent qualitative study of clinicians notes that colorectal cancer patients often exhibit more emotional distress compared to inflammatory bowel disease patients and see their ostomy as a reminder of their cancer diagnosis.^9^ Follick et al. found that 43% of patients said they did not receive enough information preoperatively and 46% did not receive enough postoperatively. Almost half of new patients with ostomies reported that if they received more information, they could have dealt with the emotional impact of the ostomy more effectively.^3^ Ostomy nurses have also been found to be incredibly helpful to patients^2^, but less is known about other beneficial interventions. The aim of this systematic review is to identify perioperative interventions created to address the psychosocial needs of new ostomy patients with colorectal cancer to improve patient quality of life. Specifically, this study aims to describe and assess the impact of practical management and psychosocial support interventions targeting colorectal cancer patients undergoing surgery with an ostomy.

## Methods

### Literature Search Strategy

We used the Preferred Reporting Items for Systematic reviews and Meta-Analyses (PRISMA) guidelines to conduct this systematic review. It was registered with the international prospective register of systematic reviews in health and social care (PROSPERO) prior to starting screening (CRD42022382142). A systematic search of Ovid MEDLINE, PsycInfo, Cochrane Clinical Trials, CINAHL, Embase, and Web of Science was conducted on September 10, 2025 by a trained research librarian on our research team. Our query aimed to assess interventions created and tested for colorectal cancer patients with an ostomy. The search terms used in our query can be found in Appendix 1.

### Inclusion Criteria

Inclusion criteria encompassed interventions used before or after ostomy surgery that aimed at reducing stressors or improving quality of life among patients with colorectal cancer who underwent surgery with a fecal ostomy. Included study types were randomized control trials, prospective cohort studies, case studies, and retrospective studies. We excluded descriptive reports of proposed interventions that were untested and those studies where specific analysis of colorectal cancer patients was not performed or not clear based on review of the results. The primary outcomes of interest for our systematic review were 1) the interventions developed and 2) patient reported outcomes, including quality of life.

### Data Extraction and Outcome Measures

We performed a title and abstract screen with three independent reviewers (SS, ARC, LB), followed by a full text review screen to determine studies that met the outlined criteria. A fourth independent reviewer (CEC) resolved any conflicts from the title/abstract or full text review screen. All screening was performed using Covidence software. The variables extracted from the included studies were country and study setting, study design type, control/comparator treatment, intervention tested, intervention duration, total participants, colorectal cancer diagnosis, type of surgery, permanency of ostomy, study inclusion criteria, study exclusion criteria, and outcomes used to determine effect of intervention (i.e., primary outcomes and secondary outcomes), and study conclusions.

### Assessment of Heterogeneity

Following data extraction, the substantial heterogeneity observed in both interventions and outcomes measures precluded the conduct of a meta-analysis. Therefore, we performed a systematic review with narrative synthesis of the main findings from the included studies.

### Assessing the Risk of Bias

Cochrane Risk of Bias 2.0 Tool, a peer reviewed tool that assesses risk of bias across 5 domains common to randomized control trials (RCT), was used to assess the RCTs for bias. Prospective cohort studies were assessed using the unmodified Newcastle-Ottawa Scale, a widely accepted tool for assessing non-randomized control trials.^10^ Bias was assessed by two study team members (ARC, CM) and all conflicts were resolved by the senior author (CC).

## Results

### Study Cohort

**Figure 1.**
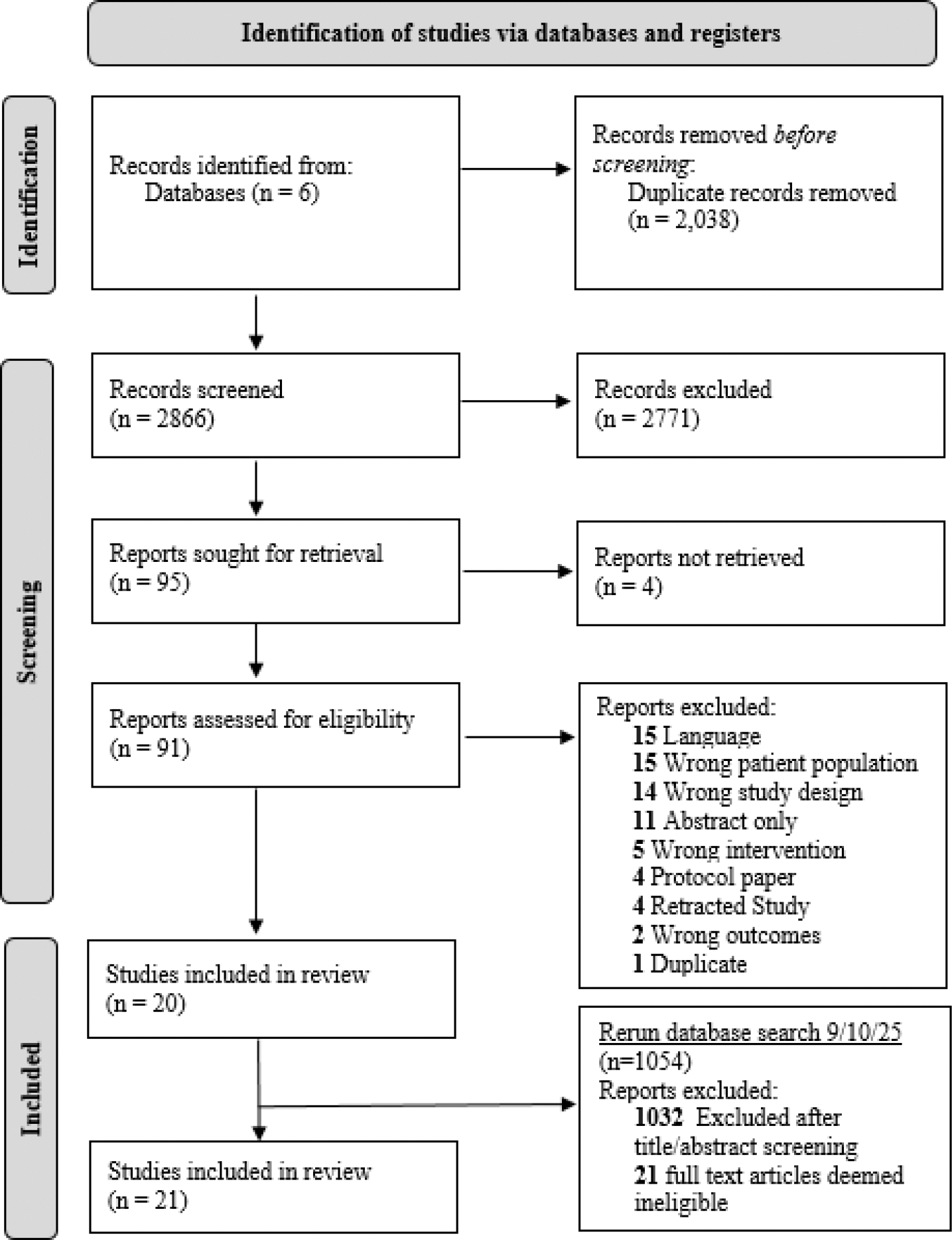
PRISMA flow diagram of the study selection process Source: Page MJ, et al. BMJ 2021;372:n71. doi: 10.1136/bmj.n71. This work is licensed under CC BY 4.0. To view a copy of this license, visit https://creativecommons.org/licenses/by/4.0/

We identified 4904 studies after a systematic search; reduced to 2866 after removing the duplicates. We eliminated 2,771 studies during abstract screening, and 4 full-text articles could not be retrieved, leaving 91 studies for full-text review. After this review, we excluded 67 studies and removed 4 due to journal retraction. Ultimately, we included 20 studies in the analysis (Figure 1) comprising 2,006 unique patients, who were typically distributed equally between intervention and control groups. The updated search resulted in 1,054 articles and recent clinical practice guidelines references were also reviewed. This list of articles was reduced to 22 articles after title and abstract screening. After full-text review, only 1 additional article met inclusion criteria.

The majority of the studies (15 of 21) were conducted in China^11–25^ with other studies conducted in Turkey^26,27^, Thailand^28^, Singapore^18^, India,^29^ and the United States^30^. Most of the studies were conducted at a single university-affiliated, tertiary care hospital. Three studies had a multicenter design including regional/rural centers.^28,30,31^ All studies evaluated quality of life as an outcome of interest [Table 1].

Most of the studies included only adult patients. Three studies excluded patients older than 70 years.^13,19,22^ Included studies considered patients with colon, rectal, or colorectal cancer confirmed either clinically or pathologically. Some studies distinguished between colon and rectal cancer patients or included a narrow cohort of only colon or rectal cancer patients. The majority of studies included patients able to read, write and speak the primary language spoken in their country. Most studies excluded patients with severe comorbidities, psychiatric disorders, poor self-care ability, and/or hearing or cognitive impairment. Some excluded patients with metastatic disease or patients with limited life expectancy (e.g., estimated survival under 3 months).^11,15,17,20^

Some studies included only patients with neoadjuvant therapy and others excluded patients who had therapies other than surgery. The type of surgeries included were abdominoperineal resection (APR) and non-restorative lower anterior resection (n-rLAR or Hartmanńs procedure). Some did not specify the type of surgery other than the inclusion of a colon or rectal resection with ostomy formation. Most of the studies included only patients with permanent ostomies; however, 1 study included only patients with temporary ostomies^11^, 3 included both permanent and temporary ostomies^18,27,30,31^, and 6 did not specify ostomy permanency. Most studies were designed to be tested in the elective surgery setting only or could be assumed to be elective cases by the preoperative delivery of the intervention [Appendix 4].

### Description of Control Groups

“Usual care” controls were not defined for all studies. Some studies described usual care controls as including routine clinical care provided by nurses and a varying number of sessions for education and support in ostomy care and follow-up visits. The description was variable and included both in person and/or virtual visits/telephone calls. One study included preoperative education and skills training provided by nursing and discharge ostomy self-care education by physicians.^22^ Some studies included preoperative ostomy site marking as a usual care control^11,18,31^ and others did ostomy marking only for the intervention group. Three provided emotional support via nurses or patient volunteers with ostomies as usual care^11,13,20^ [Table 1].

### Description of Interventions

Interventions were variable in their complexity and duration. 18 of 21 studies tested a care bundle addressing practical ostomy care, complications, and emotional/adaptation challenges. Three studies tested only one additional intervention to the routine care, such as delivery of an instructional booklet^29^, use of lavender oil for odor control^26^, or progressive muscle relaxation to reduce anxiety^31^. Most interventions included at least one type of delivery of either written or verbal ostomy self-care education. In addition, the location of the intervention was not consistently described in many studies (e.g., in-person, virtually, or over the phone). Many studies included manuals and booklets for patient education. Other studies included audiovisual resources, websites, and chat platforms or forums. Most included skills training for the patients and some interventions included training for care-partners. Some studies included practical support and education provided by patients with ostomies.

Emotional support was part of several interventions; however, the type of emotional support, structure or strategies used, and frequency was variable, or not clearly defined. Three studies included meditation or relaxation methods to address emotional challenges,^15,28,31^ such as anxiety or depression. Half of the studies included formal follow-up visits for the intervention group. These formal follow-up visits were performed over the phone, via video calls, or in-person. Other studies used chat platforms to provide emotional support and follow-up with patients on demand. One study included psychosocial and other activities: problem-solving and communication skills training, recreation activities, religious activities, and music therapy.^28^ Another study included the traditional Chinese medicine technique of foot reflexology^20^ [Table 2].

Intervention duration was highly variable ranging from a single interaction to 6 months of interactions with multiple sessions/visits. The number of sessions or visits ranged between 1 to 7. Visits were conducted in person at the clinic or at home, virtually, or by telephone. The duration of sessions was described in one study ranging from 30–40 minutes [Table 1].

### Outcomes Evaluated

As quality of life (QoL) was our outcome of interest, all studies included in this review evaluated QoL either as a primary or a secondary outcome [Table 3]. Overall, the studies included various patient reported outcomes, such as QoL, mental health, symptom burden, and engagement in social activities. Several studies also reported clinical outcomes obtained from study staff evaluations or chart reviews. The majority of studies did not define a single primary outcome. Studies that did define primary outcomes used self-efficacy^11,14,23^, self-care ability^16^, ostomy-related complications^19^, and presence of odor^26^ as their primary outcomes. One study identified 2 primary outcomes: ostomy QoL and anxiety.^31^ Seventeen of 21 studies used validated multidimensional questionnaires to evaluate QoL. Five studies used custom questionnaires or a combination of QoL scales evaluating domains such as anxiety, depression, and self-care ability. The most commonly used questionnaire to evaluate QoL was the European Organization for Research and Treatment of Cancer Quality of Life Questionnaire-Core 30 Version 3.0 (EORTC QLQ-C30 V.30), however it was only used in 4 studies.^13,19,23,25^ It is a 30-item instrument designed to measure QoL in all cancer patients. It includes five functional scales: physical, role, cognitive, emotional, and social; three symptom scales: fatigue, pain, and nausea and vomiting; a global health status/QoL scale; and six single items (dyspnea, appetite loss, sleep disturbance, constipation, diarrhea, and financial impact). The second most used survey was the Stoma Quality of Life Scale (Stoma-QOL) by Prieto et al. in 3 studies.^11,12,22^ Descriptions of each of the scales can be found in Appendix 2.

Over half of the included studies evaluated the presence of anxiety and depression among patients. Six of 20 studies simultaneously employed the Self-rating Anxiety Scale (SAS) and Self-rating Depression Scale (SDS).^14,15,17,21,24,25^ Two studies utilized the Hamilton Anxiety Rating Scale (HAM-A) and Hamilton Depression Rating Scale (HAM-D).^20,21^ Two other studies measured anxiety and depression using the Hospital Anxiety and Depression Scale (HADS).^18,30^ Two studies evaluated anxiety alone using the State-Trait Anxiety Inventory (STAI).^22,31^

Several studies evaluated outcomes related to ostomy self-care or emotional well-being. Four measured ostomy self-efficacy alone^11,18,22,23^, which is described as patient confidence in different ostomy-related activities.^32^ These studies used the Stoma Self-Efficacy Scale by Bekkers et al^33^ which includes confidence in engaging in social activities while having an ostomy. One study evaluated general self-efficacy described as an individual’s belief in their own ability to respond to a novel situation.^34^ Three studies evaluated self-care agency alone^12–14^, which is the person’s ability to engage in self-care including cognitive, psychomotor and emotional skills^35^, using the Exercise of Self-Care Agency Scale (ESCA).^36^ Other studies used non-validated scales that included various aspects of ostomy self-care, ranging from knowledge alone^29^ to a composite of domains evaluating self-care knowledge, ability, and attitudes towards ostomy care.^16,17,24,25^ Other ostomy-related outcomes evaluated were ostomy reversal rate^11^, presence of odor^26^, and ostomy acceptance.^26,27^ In 11 studies ostomy related complications were described.^11–15,19,20,24^ Seven studies included nursing satisfaction or satisfaction with the intervention as an outcome of interest [Table 4].^11,13,15,17,22,24,25^

### Study Findings:

Overall, 76.2% of the studies (16/21) reported a positive primary outcome or composite of primary outcomes for the intervention group.^11–17,19–26,29^ These studies assessed heterogeneous outcomes, with most reporting multiple measures. Of these 16 studies, 14 showed positive results across all primary and secondary outcomes, while two^23,26^ had mixed results in secondary outcomes. The remaining four studies^18,27,28,30,31^ reported no significant difference in Quality of Life between groups or mixed results when using different scales. No harm was associated with the interventions [Table 3]. Interventions showing efficacy often relied on interventions with websites, booklets, or other remote resources to provide additional support beyond usual care in a convenient way for the patient to access.

Seventeen out of the 21 studies used a single validated multidimensional QoL scale. Of these seventeen, 11 (64.7%) demonstrated statistically significant improvements in all dimensions or the overall QoL scores for ostomy patients who received supportive interventions compared to the control group.^11–15,19–22,25,26^ Two studies (11.8%) yielded mixed results with changes in only some of the dimensions.^23,31^ The remaining studies using a single validated multidimensional QoL scale found no significant score difference between intervention and control groups.^18,27,28,30^

### Bias:

Evaluation for bias revealed low to moderate risk across the majority of the included studies. The most common sources of potential bias were non-randomization of participants, lack of blinding, and the use of multiple outcome measurement scales. Most of these factors were often unavoidable due to the nature of the interventions and study designs employed. [Appendix 3]

## Discussion

Our systematic review illustrates the heterogeneity of supportive interventions designed to improve quality of life among patients with colorectal cancer recovering from surgery with a fecal ostomy. The vast majority of interventions revealed improvement in QoL for this surgical patient population when compared to a usual care control, with no harm identified in any intervention group. Of note, only one study was performed in the United States. All interventions included some type of education in ostomy care with frequent use of emotional support and remote resources to assist patients in adapting to daily life. Usual care definitions were heterogeneous with variable support in the pre- and post-operative settings.

Most studies found positive improvement in QoL outcomes from the interventions.^11–17,19–26,29^ Three studies revealed no difference in QoL between groups, but one required participants to have had the ostomy for at least 3 months^27^ and another 6 months.^28^ It is possible that the lack of improvement in QoL was due to the long period of time that elapsed from the creation of the ostomy or other treatments needed in this cancer patient population. This data suggests that the timing of delivery of the supportive ostomy intervention is important to improving patient QoL and may be most beneficial immediately prior to or following surgery.^37^ Additionally, in the study by *Lumdubwong et al.*, the intervention effect on QoL was evaluated in retrospective comparison between users and non-users finding no difference between QoL.^28^ However, the authors did find higher levels of stress and use of coping strategies. Patients experiencing higher stress levels might be more likely to utilize resources.

Three studies found no significant difference in QoL between groups.^18,30,31^ In these studies, the control and intervention groups received standard care that often includes stoma site marking and education provided by ostomy nurses in both inpatient and outpatient settings. This robust control likely led to a smaller difference between groups. One study showed increased self-care ability over time for both groups. A study by Krouse *et. al*, revealed that higher intervention attendance was associated with improvement in quality of life domains and anxiety.^30^ In another study by Lim et al. the intervention group demonstrated lower anxiety levels and higher ostomy acceptance compared to the control groups.^18^ These effects are consistent with other studies including interventions aimed at reducing anxiety and depression among patients living with an ostomy. Targeted interventions designed to address psychological outcomes are effective when combined with practical care interventions.

All interventions incorporated educational content aimed at enhancing ostomy care ability in this study. A recent systematic review by Danielsen et al. found that structured patient education significantly improved health-related QoL, proficiency in ostomy management, and psychosocial adjustment.^38^ In addition, educational programs that involve multimedia-based interventions and remote resources have proven more effective than conventional education according to recent studies including a systematic review.^39,40^ These could relate to the accessibility of consistent and convenient content of these intervention that can improve intervention fidelity and scalability. Prioritizing patient education along with communication, involving caregivers to increase mutuality^41^ in care and support in the early stages of ostomy adaptation is crucial to prevent negative emotions^42^ that can have long-lasting impact. These impacts may extend beyond the patient’s physical and mental health, affecting employment, finances^43^, and personal relationships, and potentially leading to caregiver burden.^44^ Overall, it seems that most control and intervention groups included preoperative stoma marking and access to ostomy nurses, which have been previously proven to be helpful^3,45^ but then added additional novel resources, such as digital interventions or multimedia, which appear to be beneficial for improving QoL. Interventions were also often bundles, rather than one discrete, single intervention. These bundles frequently included continuity with support from nurses or other patients, which highlights that ongoing support and personal contacts for encountered issues may be the most beneficial to ostomy patients. This is consistent with the benefit of ostomy nursing support in terms of QoL.^3,45^

### Limitations and strengths

The studies were almost all performed outside the United States, which may limit generalizability in the United States. This reveals a great opportunity for surgeons to improve the outcomes of surgical patients through targeted interventions in the United States. Moreover, most studies excluded vulnerable populations such older adults^7^, those with cognitive impairment, disability, lack of reading or written comprehension, and high-risk comorbidities. For example, patients from diverse backgrounds and socioeconomic statuses may benefit from ostomy education, as limited education can lead to wasted supplies and increased costs.^43,46^ Future research should focus on evaluating the effectiveness of supportive care interventions among these vulnerable populations. Most interventions were studied in elective surgical cases to allow enrollment in the study. This limits generalizability for patients undergoing ostomy surgery on a more urgent or emergent basis. In addition, comparing the effectiveness of the interventions in a metanalysis was not possible due to the heterogeneity of the interventions/controls and different outcomes used to evaluate QoL. There was also heterogeneity in the description of usual care controls, with some patients receiving extensive resources and others receiving minimal teaching and support. This is consistent with the variability in resources, such as trained ostomy nurses, that is seen across the United States.^47^ Future studies evaluating interventions should continue to provide detailed descriptions of the care received by the control group to allow for comparison with the intervention group. This can help providers determine if new interventions add significant benefit outside of the interventions already found to have proven benefit, such as ostomy nursing support.^3,45^ Standardization and use of validated QoL surveys will also be beneficial to make comparisons across studies. Surgical societies could help promote some of these validated surveys to aid researchers in selecting tools.

## Conclusion

Overall, the majority of interventions aimed at improving the quality of life of colorectal cancer patients with fecal ostomies following surgery demonstrated positive benefits. Demonstrated benefit must be taken in the context of most studies being conducted outside of the US and excluding vulnerable populations, including older adults, patients with multimorbidity, cognitive impairment, and those with discordant native language, which limits generalizability. Further research is needed to evaluate the efficacy of these interventions in broader patient populations, and to facilitate the implementation of effective strategies to enhance patient quality of life across health systems. This information is crucial for clinicians providing direct patient care, researchers designing future studies, and healthcare policy makers developing guidelines and allocating resources to this patient population. By addressing these gaps in care and current research, we can develop comprehensive and scalable strategies to improve the overall care and outcomes for colorectal cancer patients with ostomies.

## Supplementary Material

1 Appendix 1. Search terms used for systematic review query

2 Appendix 2. Description of Multidimensional Quality of Life Scales

3 Appendix 3. Bias Assessment

4 Appendix 4. Inclusion criteria

## Figures and Tables

**Table 1.** Study setting, design, and description of usual care and interventions.

	Study	Country	Study Setting	Study Design	Usual care (Control) Description	Intervention Description	Intervention duration/number of sessions	Int N =	Con N =	Total N=
1	Su X, et al. 2021	China	Multicenter (4), tertiary care, university affiliated. Each hospital has full-time enterostomal therapist and wound care RN w >10yrs experience	RCT	- Preoperative stoma site marking - Post-operative assistance with appliance change by an ET or WOCN. - Education: preventing complications, medications, diet, recovery. - Visits by volunteers from the stoma association to share their experiences on stoma self-care. - Patients could request additional telephone or outpatient follow-up with nurses.	Continue Care Bundle (CCB): - Self-management manual: surgery and stoma type details, stoma care, appliance change, stoma dilatation, self-image, diet, rest and activity, work, sexual activity, emotions. - Scheduled telephone and stoma outpatient clinic follow-up with nurses.	One year-participants enrolled until 12/2015 and study participation ended when stoma was reversed, or at close of study in 12/2016 if they hadn’t been reversedyet. Sessions included one telephone follow up, and one outpatient follow up	60	64	124
2	Zhang X, et al. 2020	China	Multicenter (3), tertiary care, university affiliated.	RCT (Double blinded)	- Preoperative preparation and postoperative rehabilitation by nurses - Education: diet, medication, recovery and ostomy care. - After discharge, routine telephone follow-ups and outpatient visits performed by the same nurse. - Health Education Handbook for Hospital Home Care for Patients Undergoing Permanent Enterostomy (given to control group at the end of the intervention)	Timing it right (TIR) 5 stages of intervention: 1. Diagnosis: - Education: Diagnosis, treatment, & team care - Emotional support & establishing relationships 2. Perioperative (Stabilization) - Education: preop preparation - Emotional support 3. Discharge Preparation - Education: ostomy care, complications - Rehabilitation plan: medication, diet, exercise - Emotional support 4 Adjustment (Discharge-3 months) - Education: ostomy care, healthy lifestyle, acceptance and adaptation, complications - Emotional support: communication and organizing social gatherings 5. Adaptation (3–6months) - Education: health education, return to society - Emotional support: ostomy/change in defecation & self-image acceptance	First month: 2 phone calls/week, and one clinic visit every 2 weeks. Second month, one phone call/week and one clinic visit every 2 weeks. Third month, one phone call every two weeks and one clinic visit every three weeks. Fourth month, one phone call per month and one clinic visit per month. Each visit was 30–40 minutes.	60	59	119
3	Andrews GR, Sharma A. 2018	India	Multicenter (2), tertiary care, university affiliated.	Quasiexperiment al Time- series (nonrando mized, prepost intervention)	Routine nursing (no further description provided)	Instructional booklet (IB) given to patients: colorectal cancer, colostomy care, appliance change, diet, self-care, daily life, sexual health, travel and physical activity, identifying complication and emotional coping.	Pretest, pre interventi on. Post test given at 30, 60, 90 days	100	100	200
4	Cengiz B, et al. 2020.	Turkey	Multicenter (3), tertiary care, university affiliated.	NRCT (Control: different hospital)	- Routine clinical care - Outpatient and telephone follow-ups - Intervention booklet (given to control group at the end of the intervention)	Educational Booklet + 6 Monthly Home Visits: 1. Meeting the family or care partners, home environment evaluation 2. Stoma appliances and stoma care 3. Daily life activities with stoma 4. Coping with chemotherapy and adverse effects 5. Stoma complication prevention and treatment 6. General education, addressing specific issues	6 months. 6 sessions	30	31	61
5	Cheung YL, et al. 2003.	Hong Kong	Multicenter (2), one major referral center and one regional center.	RCT	- Preoperative stoma site marking - Education (written and skills training): preparation for surgery, stoma care and appliances. - Postoperative inpatient wound and stoma care - Surgical outpatient and stoma clinic follow-up visit 4–6 week after discharge.	Progressive muscle relaxation therapy (PMRT): - Information and training session - Recorded audio instructions - Subjects kept a log of practice sessions - Follow-up every 2 weeks after discharge	baseline, 5 weeks, 10 weeks	32	32	64
6	Duluklu B, et al. 2019	Turkey	Multicenter (2), Public Training and Research Hospitals General surgery clinics.	RCT (Single blinded)	- Routine stoma care for 1 month - Education: stoma care and nutrition information - Handoff: nutrition recommendations to reduce odor from uncontrolled gas and stool output	Use of lavender essential oil in the ostomy bag to prevent odor: - Education: application of lavender oil during appliance change - One month supply of the oil	1 month	15	15	30
7	Huang L, et al. 2018.	China	Single center, tertiary care, university affiliated.	RCT	- Routine nursing (including psychological support by nurses on prognosis and dealing with negative emotions after surgery) - Education: stoma care and diet. - Telephone follow-up once a week.	Continued nursing: - Routine nursing prior to discharge - 1 training session with patients and caregivers before discharge. - Once a week home or telephone follow-ups with designated health care professional discussing diet, sleep, defecation, stomal adaptation after discharge. - Video education: stoma care and appliance change	1 day before discharge - 4.5 months postop (1 session before discharge, once a week at home of telephone follow up)	60	60	120
8	Huang Q, et al. 2021.	China	Single center, tertiary care, university affiliated.	NRCT (Control: admitted earlier Intervention: admitted later)	- Routine nursing care - Ostomy care instructions included in discharge summary - Regular follow-up visits to hospital and Routine telephone follow-up	Online based continuous nursing care: - Established nursing team - Education: ostomy care, dealing with negative emotions and adapting to daily life. - Access to group chats with patients sharing successful self-care experiences and new self-care methods/products and moderated by nurses - Monthly video calls to discuss challenges/soluti ons - Access to public website with tips and videos	6 months after discharge	62	57	119
9	Jiao H, et al. 2020.	China	Single center, general hospital, military affiliated.	RCT	- Routine nursing: conventional nursing care with monitoring of vital signs - Education: stoma self-care, diet, general health and discharge instructions	Humanistic group: - Preoperative psychological intervention - Preoperative stoma site selection (agreed by surgeon and patient together) - Postop targeted nursing (colostomy care, diet adjustment, preventing stoma-related complications) - Rehab nursing (weekly telephone follow-up and monthly home follow-up with education on stoma-related knowledge and self-care methods) - Social support (encourage to reach out to friends and family) and discussions with ostomates - Yoga and meditative relaxation method for those with negative emotions.	Preoperati vely then postoperat ive had weekly telephone follow-up, home follow-up once monthly, and class organized once every 3 months	45	45	90
10	Jin Y, et al. 2021	China	Single center, tertiary care, university affiliated.	NRCT (Control: admitted earlier Intervention: admitted later)	Routine nursing (no further description provided)	FOCUS-PDCA: - Find: problems during colostomy clinic - Organize: using healthcare team to help improve self-care ability - Clarify: use of the ESCA self-care ability scale to assess patient ability - Understand: fishbone diagram to evaluate possible reasons for low self-care ability - Select: four root causes and implement countermeasures to combat them - Plan: set the target goals for improvement in the Exercise of Self-care Agency scale (ESCA) score - Do: continuous nursing via telephone follow-up, family visit, colostomy fraternity - Preoperative: stoma site marking, education, skills training, booklet and psychological guidance - Postoperatively: Education and skills training - Check: repeat ESCA at 1 week & 1 month - Action: evaluate results and intervene if needed	Self-care evaluated at 1 week and 1 month after surgery	80	80	160
11	Li L, et al. 2021.	China	Single center, tertiary care, university affiliated.	Controlled Trial (does not specify randomizati on)	Routine nursing (no further description provided)	Predictive nursing: - Active physical examinations - Education (with patients & family): colorectal cancer, stomas care, appliances, diet. Including skills training. - Emotional state evaluation and support by nurses - Multimedia: stoma and self-care. Skills training with stoma in place, selected appliances. - Printed information.	Does not specify number of sessions or timeline. Apparently measured preop and postop before discharge. Measured after 1 month.	80	50	130
12	Lim SH, et al. 2019	Singapore	Single center, tertiary care public hospital. university affiliated.	Pilot RCT (Single blinded to the data collectors)	- Preoperative stoma site marking - Education: stoma education at 3–5 postop including demonstrations by stoma nurse, if needed another session after discharge	STOMA psychosocial intervention: - Preoperative in-person psychoeducation al session - Educational booklet - 5 phone follow-ups (1 preop, 4 postop)	Preop until 4 months after discharge. T1: enrollmen t preop. T2: Day of discharge, T3: 4 weeks after discharge and T4: 4 months after discharge	29	24	53
13	Liu H, et al. 2019.	China	Single center, university affiliated.	RCT	- Routine nursing: nursing education before and after surgery - Precautions after discharge - Follow up at 1 or 3 months	Continuous care three-in-one model involving hospitals, communities, and families: - Hospital: ostomy nurses provide education, skills training and reassurance. Monthly ostomy meetings - Community: registration card system with clinical summary, training for nursing staff and doctors. - Families: designated coordinators	Preop- 3 months postop (Monthly ostomy care visits, telephone follow up once a week)	40	40	80
14	Liu Y, Ni L. 2021.	China	Single center, university affiliated.	RCT	- Routine nursing - Health education - Psychological and family counseling - Telephone Follow-up every 3 weeks.	Continuous nursing intervention through an online platform (WeChat or QQ): - Initial health and psychosocial assessment. - Online education (written and videos): self-care skills, foot reflexology, diet, physical activity. - Patients were encouraged to communicate with each other for mutual support.	Does not specify	63	63	126
15	Lumdub wong A, et al. 2014.	Thailand	Multicenter (3), National Cancer Institute, Tertiary care Hospital, Rural Cancer Center. University affiliated.	Retrospective Comparative descriptive, users of the resources vs nonusers	- Routine nursing only and did not participate in any of the sessions offered by the hospital.	Friendship Therapy Centers offered sessions and activities: - Group sessions about illness experiences - Education programs - Stress management activities - Problem-solving and communication skills trainings - Recreative activities: handcrafts, traveling, painting. - Religious activities - Music therapy	Does not specify	30	57	87
16	Wang S, et al. 2021.	China	Single center, tertiary care, university affiliated.	RCT	- Routine nursing: vital signs measurement, medication guidance, education.	Targeted psychological nursing interventions: - Preoperative stoma site marking - Preoperative education (patient and family): stoma care, rectal cancer, diet and nutrition education. - Postoperative (before discharge): individualized psychological intervention, self-care education and follow-up visits list. Provided additional stoma care information accessible through internet.	1 year recruitment (unspecified duration of interventi on). Pre and post operative	60	60	120
17	Xia L. 2020.	China	Single center, tertiary care, university affiliated.	RCT (Single blinded)	- PO day 1: inpatient ostomy care and skills training by nurses - Day before discharge: education (self-care, appliance change, diet, lifestyle) provided to family and the patient by the doctor	- Preoperative: ostomy care manual and video education, ostomy nurse answered questions. - After discharge: remote communication patient and caregiver with ostomy therapist (WeChat, blog, QQ, telephone, etc.). Caregiver recorded changes in mood or complications	3 months after discharge	81	74	155
18	Xu S, et al. 2018.	China	Single center, tertiary care, university affiliated.	RCT	Routine nursing (no further description provided)	Self-efficacy intervention: sessions including education, skills training, social and psychological support. Content per session was based on patients’ feedback.	Unclear - 3 months postop (7 sessions: 1 st month - once a week, 2nd - twice a month, 3rd - once)	20	28	48
19	Xu W, et al. 2020.	China	Single center, tertiary care, university affiliated.	Retrospective	- Routine care and conventional post-discharge home care and routine recovery monitoring. - Education: appliances and basic stoma care. - Health manuals	Comprehensive continuing care: - WeChat group with attending physicians and nursing staff to discuss the best management. Second group with patients and nurses to discuss recovery, emotional and self-care concerns. - Nursing plan for each patient, including tailored psychological support provided by nurses. - Education: diet, exercise, sleep hygiene. - Offline peer education.	1 month postop	30	30	60
20	Yu S, et al. 2021.	China	Single center, tertiary care, university affiliated.	RCT	Routine nursing (No preoperative stoma site marking)	Comprehensive care: - Preoperative stoma site marking - Close vital sign monitoring - Education and skills training: ostomy care, appliance changes, self-care. - Psychological support: Encouraging patients to express feelings, building confidence. - Reassurance for self-care and social participation.	One day preop - discharge day (One session preop, undefined number of sessions postop during admission)	30	30	60
21	Krouse, et al. 2024	United States	Multicenter, tertiary care	RCT	Routine nursing (no further description provided)	A telehealth curriculum led by ostomy nurses and trained peer ostomates including didactic and guided discussion - Session 1: information on ostomies - Session 2: psychosocia l issues - Session 3: healthy living (travel, diet and exercise) - Session 4: care-partner education session - Session 5: Booster session led by attendees	Five group sessions lasting 1.5 hours each	106	110	216

RCT: Randomized Controlled Trial; Preop: Preoperative; Int: Intervention Group; Con: Control Group.

**Table 2.** Frequency of Interventions

	Study	Ostomy Site Marking	Education	Manuals/Booklets	Practical training/support			Emotional support			Additional Follow-ups			Video/ Audio	Chat/ Forums	Digital/ Website	Meditation/Relaxation	Other
					Nurses/Staff	Ostomates	Family/Friends	Nurses/Staff	Ostomates	Family/Friends	Telephone	Videocall	In-person					
1	Su X, et al. 2021	+	+	*	+	+			+		*		*					
2	Zhang X, et al. 2020		+	*	+			*	*	*	*		*		*			
3	Andrews GR, Sharma A. 2018		*	*														
4	Cengiz B, et al. 2020.		*	*	*		*	*		*								
5	Cheung YL, et al. 2003.	+	+	+	+						*		*	*			*	
6	Duluklu B, et al. 2019		+	+														Lavender oil
7	Huang L, et al. 2018.		+		*			+			*			*				
8	Huang Q, et al. 2021.		*		*	*		*	*			*			*	*		
9	Jiao H, et al. 2020.	*	+		*			*	*	*	*		*				*	
10	Jin Y, et al. 2021		*		*			*	*	*	*		*		*			
11	Li L, et al. 2021.		*	*	*		*	*						*				
12	Lim SH, et al. 2019	+	+	*	+			*			*							
13	Liu H, et al. 2019.		*		*		*	*		*	*							
14	Liu Y, Ni L. 2021.		+					+	*					*		*		Foot reflexology
15	Lumdubwong A, et al. 2014.		*		*	*		*	*								*	Problem-solving and communication skills training, recreative, religious activities, & music therapy
16	Wang S, et al. 2021.	*	+		*		*	*								*		
17	Xia L. 2020.		+	*	*		*	*		*	*			*	*			
18	Xu S, et al. 2018.		*		*			*										
19	Xu W, et al. 2020.		+	+	*	*		*	*						*			
20	Yu S, et al. 2021.	*	*		*			*										
21	Krouse, et al. 2024		*		*	*	*	*	*	*		*				*		

* Intervention Group Only

+ Usual Care (Control) & Intervention Group

**Table 3.** Study Results

	Study	Outcomes	Results	Conclusion
1	Su X, et al. 2021	Primary: Self-efficacy Secondary: QoL, treatment satisfaction, stoma reversal rate, stoma complications.	The intervention group had significantly improved self-efficacy (F = 11.88, p <0.001), quality of life (F = 17.99, p < 0.001) over time, satisfaction (t = 4.08, P < 0.001), and outcomes of stoma reversal (χ2 = 5.93, p = 0.015) and reduced the incidence of complications (p < 0.05).	Evidence-based continuing care bundle can be an effective method to improve the health outcomes among colorectal cancer patients with temporary stomas.
2	Zhang X, et al. 2020	Unclear single primary: Self-efficacy, stoma QoL, resilience, stoma complications.	No difference between groups baseline characteristics. At 3 or 6 months after discharge, the psychological resilience and quality of the intervention group were significantly higher than those of the control group (t = 4.158/7.406, t = 4.933/8.611, p < 0.05). At the time of discharge and at 3 or 6 months after discharge, the self-care ability of the intervention group was significantly higher than that of the control group (t = 1.543/3.656/6.273, p < 0.05). Incidence of complications at 3 and 6 months was lower in the intervention group (p <0.05)	TIR theory-based hospital-home holistic nursing intervention effectively enhances the psychological resilience and self-care ability of patients with permanent enterostomy, reduces complications and improves quality of life, especially in the rehabilitation stage after discharge.
3	Andrews GR, Sharma A. 2018	Unclear single primary: Knowledge, attitude, problems with stoma, coping skills	Information booklet on colostomy care significantly increased colostomy patient’s knowledge (87.6 vs 52.1), increased positive attitude (9.9 vs 9.3), decreased physical (44.5 vs 50.2) and psychosocial (50.2 vs 60.6) problems and improved emotion (35.9 vs 31.7) and problem focused (6.0 vs 4.7) coping scores at 90 days (p<0.001).	Information booklet was found to be effective in enhancing knowledge, positive attitude and decrease physical and psychosocial problems and enhance adoption of better coping. It was also found to be acceptable and useful as expressed by colostomy patients.
4	Cengiz B, et al. 2020.	Unclear single primary: Stoma QoL, ostomy adjustment and complications Secondary: Compliance with stoma care, hospital visit due to complication and treatment costs.	QoL was not statistically significant difference between groups pre or posttest at 6 months. However, there is a significant increase seen in pre vs posttest in the experimental group alone (22.08 to 24.17, p <0.001). For ostomy adjustment, there is a non-statistically significant difference between groups pre or posttest. But there was a significant difference between groups and increase within the experiment group when comparing pre to post test (27.08 vs 27.7, p<0.001/23.79 vs 23.13, p<0.01). Complication index was significant difference pre and post for both groups (24.81 to 20.62, p<0.03/26.25 to 30.79, p<0.01) and between groups (20.6 vs 30.79, p <0.01) with less complications in the intervention group. Experimental group showed higher compliance with stoma care, lower hospital visits due to complications, and reduced treatment costs compared to the control group.	No difference in self-care scores or quality of life was observed between groups. But higher compliance with stoma care and lower readmissions with reduced treatment costs. The study demonstrates the value of long-term follow-up by stoma nurses. Authors recommend creating hospital-based home care teams for 6-month post-discharge follow-up of individuals with stomas.
5	Cheung YL, et al. 2003.	Two primaries: Stoma QoL and anxiety	No significant difference between groups in disease specific QoL [F (1, 57) = 2.63, p = 0.01]. However both groups showed improvement in QoL over time [F (2, 56) = 35.96, p < 0.001]. There was a significant improvement in WHO QOL BREF outcomes in intervention group compared to control and the effect also increased over time [F (2, 56) = 97.63, p < 0.001]. Significant difference in STAI between groups with lower anxiety in intervention group (F= 8.99, p <0.01) effect more pronounced at ten weeks than five weeks.	Progressive Muscle Relaxation Therapy (PMRT) should be offered to patients undergoing stoma surgery.
6	Duluklu B, et al. 2019	Primary: odor (“Do you think odor is a problem for you related to your colostomy?”) Secondary: QoL and ostomy adjustment	All but one control patient reported odor issues at baseline. Post-intervention at 1 month, all intervention group patients had no odor problems, while all 14 control patients continued to experience odor issues at 1 month. Intervention group had statistically significant increase in overall life satisfaction, work/social function, sexuality/body image, and general quality of life compared to control group (increase of 26.6 ± 4.03 vs 4.8 ± 24.358, F=11.555; p < 0.05); no difference in stoma function between the two. For ostomy adjustment, the intervention group had a statistically significant increase in acceptance, social engagement, and general ostomy adjustment compared to the control group. The control group had lower anxiety/preoccupation and anger.	Use of lavender essential oil in the colostomy bag as a measure to decrease odor led to perceived decrease in odor and improvement in QoL and ostomy acceptance compared to patients doing routine practices
7	Huang L, et al. 2018.	Unclear single primary: Stoma complications, self-care ability and quality of life. Secondary: satisfaction with treatment.	Statistically significantly lower rates of stomal bleeding, stomal stenosis, peristomal allergic dermatitis, stomal edema, and stomal abscission in the intervention group compared to control group. Significantly higher self-care ability (136.38 vs 105.01), significantly higher QoL scores (73.24 vs 54.23), and higher satisfaction with treatment (88.3% vs 40.3%) in the intervention group compared to control (all p<0.001) at 4.5 months after surgery.	Continuing nursing care following Miles’ operation with ostomy can lead to increased patient self-care ability, improved quality of life, and reduced incidence of complications
8	Huang Q, et al. 2021.	Primary: Self-efficacy Secondary: Self-care ability, QoL, depression and anxiety, and stoma complications.	Self-efficacy score improvement in the observation group after the intervention was significantly higher than the control group (18.97 to 23.18 vs 19.14 to 21.06, p <0.001). Self-care ability score improvement in the observation group was significantly higher than the improvement in the control group (98.93 to 139.72 vs 99.79 to 117.28, p <0.001). SF-36 scores (QoL) were significantly higher in every dimension for the observation group compared to the control group after the intervention. Observation group had significantly lower SDS and SAS scores than the control group after intervention (47.96 vs 52.07 and 49.83 vs 54.38, p <0.001) Complication rate in the observation group was significantly lower than the control group (4.84 vs 15.79, p = 0.048)	Online training based continuous nursing care can effectively improve the self-care ability and self-efficacy for rectal cancer patients and also contribute to improve quality of life and psychological status with reduction in complication rate
9	Jiao H, et al. 2020.	Unclear single primary: negative emotions, self-care ability, general comfort level, happiness level and QoL, complication rates and nursing satisfaction.	Significantly greater decrease in SAS and SDS scores in humanistic group compared to control. Significantly greater increase in self-care ability (67.36 to 87.57 vs 68.83 to 75.4, p <0.001) and general comfort scores in humanistic group. Of the domains of the happiness score, the positive affect and subjective well-being scores were higher and negative factor was lower in humanistic group than control group. Significantly greater increase in QoL scores in humanistic group compared to control (67.10 to 84.24 vs 68.73 to 76.42, p = 0.001). Significantly lower total complication rate in the humanistic group (2.22% vs 17.78%, p <0.001) and higher satisfaction rate than control (95.56% vs 71.11%, p <0.05)	Humanistic nursing care for colon cancer patients with stomas can improve negative emotions, self-care ability, general comfort levels, happiness levels, QoL, patient satisfaction with care, and decrease complication rates
10	Jin Y, et al. 2021	Primary: Self-care ability	Both control and observation groups improved their self-care ability scores from 1 week to 1 month. The observation group had significantly higher self-care scores at 1 week (61.5 vs 39.09, p <0.01) and 1 month (83.13 vs 60.15, p <0.01) than control.	FOCUS-PDCA can be applied to self-care management of rectal cancer patients with colostomy to improve patient self-care ability
11	Li L, et al. 2021.	Unclear single primary: Surgical indicators, self-care abilities, nutritional status, mental health, postoperative recovery, complications, and nursing satisfaction	Surgical indicators were shorter, self-management ability scores were higher, nutritional indexes were higher, and anxiety and depression scores were lower in the predictive nursing group compared to the control group (all p<0.05). Recovery effective rates 76% intervention vs 40% control. Complications: 3% vs 15% and satisfaction 78% vs 38%, respectively.	Predictive nursing was better in every outcome compared to normal nursing. For patients with rectal cancer undergoing colostomy formation, the implementation of predictive nursing can improve patient’s mood and self-management ability.
12	Lim SH, et al. 2019	Unclear single primary: Stoma care self-efficacy, acceptance of stoma, stoma proficiency, length of hospital stay, and anxiety and depression and QoL.	No significant group differences were found in QoL at T2 (F = 1.16, p = 0.35), T3 (F = 1.37, p = 0.22), and T4 (F = 1.57, p = 0.14). However, stoma acceptance was higher in the intervention group at T3 (F = 2.17, p = 0.003) and T4 (F = 8.42, p = 0.006). No significant differences were found in stoma care self-efficacy at T2 (F = 0.75, p = 0.70), T3 (F = 1.56, p = 0.15), and T4 (F = 0.89, p = 0.57), although the intervention group showed a greater overall increase. Anxiety scores significantly decreased in the intervention group from T3 to T4 (p = 0.01). Overall, both groups showed improvements in QoL and reductions in anxiety and depression from T2 to T4.	The pilot study of the STOMA psychosocial intervention program shows promising results for colorectal cancer patients with stomas, particularly in increasing stoma acceptance. However, it did not significantly improve stoma self-efficacy, quality of life (QoL), or other outcomes. The authors recommend further investigation through larger, full-scale studies to better understand the program’s effectiveness.
13	Liu H, et al. 2019.	Primary: Ostomy complications Secondary: QoL	Complication rate was higher in the control group than in the intervention group, especially in terms of skin complications and mucosal separation at 1 and 3 months. Quality of life (QoL) did not show statistically significant differences at discharge and 1 month, but it was higher in the intervention group at 3 months.	Continuous nursing model decreased complications and increased QoL in patients with permanent colostomies.
14	Liu Y, Ni L. 2021.	Unclear single primary: QoL, depression and ostomy complications	Before the intervention, there was no significant difference in QoL score between groups (p<0.05). After intervention, the study group QoL significantly improved compared to control group. Depression score in the study group decreased. The incidence of complications in the study group (7.84 %) was significantly lower than that in the control group (23.72 %) (p<0.05)	The application of continuous nursing intervention of network interactive platform in patients with colostomy after colorectal cancer surgery can significantly relieve their anxiety and depression, improve their self-care ability and quality of life and reduce the incidence of complications, which is worthy of clinical application.
15	Lumdubwong A, et al. 2014.	Unclear single primary: perceived stress, stress appraisal, coping functions, and QOL	Both groups reported moderate perceived stress scores, with the Friend Therapy Group (FTG) slightly higher. Stress appraisal was similar in both groups, ranking challenge, harmful, and threat respectively. The FTG group used more problem and emotion-focused coping, but the proportions were similar in both groups. No significant differences were found in perceived stress, stress appraisal, coping, and QOL between groups (Hotelling’s T2 =.09, p=.34). However, the FTG group used three coping strategies more: acceptance responsibility, planful problem solving, and positive reappraisal (p<0.05).	There was no effect from Friend Therapy Groups on stress, coping and adaptational outcomes. FTG had higher levels of perceived stress, which lead to the conclusion that people with higher levels of stress looked for more support. Additional coping strategies were found in FTG groups.
16	Wang S, et al. 2021.	Unclear primary: QoL, Anxiety, Depression, defecation characteristics, nursing satisfaction, sleep and employment and empowerment levels.	Baseline scores and characteristics were similar between the groups. At the end of the study, the SF-36 quality of life scores in the intervention group were higher than they were in the control group (P < 0.05). Intervention group had lower SAS and SDS scores (P < 0.05). Significant improvements were also seen in HAMA and HAMD scores in favor of the intervention group, with scores of 2.64 vs. 5.03 (HAMA) and 5.67 vs. 9.14 (HAMD) (P < 0.05). The control group experienced more defecation discomfort and stoma complications (19 vs. 7, P < 0.05). The intervention group had a lower PSQI sleep score (8.04 vs. 5.01, P < 0.05) and longer sleep duration (6.76 vs. 7.91 hours, P < 0.05). Employment and empowerment levels were higher in the intervention group.	Application of psychological nursing interventions for patients with rectal cancer stomas can relieve their unhealthy moods, improve their quality of life, and increase their level of satisfaction with the nursing care. It is worthy of widespread clinical application.
17	Xia L. 2020.	Unclear single primary: anxiety, self-efficacy, QoL, nursing satisfaction.	Pre-surgery anxiety levels were similar between groups. Three months post-discharge, the control group had significantly higher STAI scores (p < 0.0001). Self-efficacy scores were initially similar but significantly higher in the experimental group at 1 month (83.40 vs 74.56, p < 0.0001) and 3 months (111 vs 81, p < 0.0001) post-discharge. At 3 months, the intervention group had significantly higher scores in all QoL dimensions (p < 0.05) and had lower stomal stenosis frequency (p < 0.001). Patient satisfaction was higher in the continuous care model group (4.15 ± 0.21 vs 3.97 ± 0.45, p < 0.001). The average score of satisfaction of patients receiving the continuous care model was 4.15 ± 0.21, whereas it was 3.97 ± 0.45 for patients receiving the control model (continuous care model vs. control: t = 3.236, df = 153, p < 0.001).	In comparison with colostomy patients who were treated with routine care, the patients receiving the continuous care model had significantly better physical and psychological outcomes and suffered fewer colostomy complications. Also, patients reported a high degree of satisfaction with the continuous care model. Authors concluded that all patients in this population should be treated with the continuous care model.
18	Xu S, et al. 2018.	Primary: Self-efficacy effect on QoL	Self-efficacy score was not significantly different at 10 days between groups but was higher after 1 month and 3 months. With the highest difference at 3 months in favor of the intervention group (91.15 ± 10.708 vs 62.429 ± 12.630 p=0.000). The EORTC QLQ-C30 scores of the intervention group indicated that the score of each dimension was higher than that of the control group, except for the three indexes regarding dyspnea, constipation, and economic difficulties; the differences between the two groups for all other dimensions were statistically significant.	Clinical nursing intervention based on self-efficacy theory could improve self-care abilities and the quality of life for these patients through self-efficacy nursing interventions.
19	Xu W, et al. 2020.	Unclear single primary: Anxiety, depression, self-care ability, QoL.	No significant differences were found between groups at baseline (all p>0.05). After one month, the continuing care group showed lower SAS/SDS scores, and improved self-care skills compared to routine care (all p<0.05). Both groups improved in all QoL scores after the intervention, with the continuing care group showing greater increase (p<0.001). Continuing care patients reported significantly higher nursing satisfaction (96.66% vs. 76.66%, P< 0.05) and a lower rate of ostomy complications (6.67% vs 26.70% p=0.038).	Comprehensive continuous nursing is conducive to the rehabilitation of early post-discharge patients with colostomy caused by rectal cancer and can effectively improve psychological status. Therefore, it is worthwhile being widely used and promoted in clinical practice.
20	Yu S, et al. 2021.	Unclear single primary: Recovery of GI function, Self-care ability, Pain 3 days after surgery, Anxiety, Depression, complications, knowledge about ostomy care, QoL, nursing satisfaction.	The first time of exhaust, food intake and the recovery of bowel sound in the research group were markedly earlier than those in the control group. The research group had a notably lower incidence of postoperative complications (3.3% vs 26.6% p<0.05), lower SAS and SDS scores at discharge. Intervention group had higher average self-care ability than the control group, as well as higher quality of life scores in all dimensions (p<0.05) and nursing satisfaction.	Comprehensive care intervention can promote postoperative recovery of colorectal cancer patients after colostomy, relieve their negative emotions, reduce postoperative complications, improve quality of life and nursing satisfaction, which is worthy of promotion in clinical practice.
21	Krouse, et al. 2024	Multiple primary end points: patient activation measure, Change in ostomy management self efficacy and City of Hope QoL-ostomy	No significant improvement in patient activation measure (4.0 versus 2.9) at 6 months. No significant difference in other end points. However, higher attendance of sessions was associated with post-session improvement in self-efficacy (p<0.05), two quality of life domains (p<0.05), and anxiety (p=0.01).	There is no clear benefit with the intervention. However, fidelity of the intervention might impact efficacy of the intervention.

QoL: Quality of Life; TIR: The theory of Timing it right; WHOQOL-BREF: World Health Organization Quality of Life – BREF; STAI: State-Trait Anxiety Inventory; SAS: Self-Rating Anxiety Scale; SDS: Self-Rating Depression Scale; HAMA: Hamilton Anxiety Rating Scale; HAMD: Hamilton Depression Rating Scale; EORTC QLQ-C30: European Organization for Research and Treatment of Cancer Quality of Life Questionnaire-Core 30

**Table 4.** Outcomes Measured and Study Timepoints

	Study	Quality of Life	Anxiety	Depression	Ostomy self-care	Ostomy complications	Ostomy acceptance	Nursing satisfaction	Other outcomes	Timepoints
1	Su X, et al. 2021	Stoma Quality of Life Questionnaire (Stoma-QOL)			Stoma Self-Efficacy Scale (Bekkers et al) and Number of days to stoma care proficiency	Incidence proportion of each type of complication, collected from medical record (peristomal dermatitis, mucocutane ous separation; stoma prolapse, stenosis, retraction; parastomal incision infection, hernia, granuloma)		5-point satisfaction scale (Intervention satisfaction)	Stoma reversal	Baseline (day of discharge), 4 weeks and 12 weeks after surgery.
2	Zhang X, et al. 2020	Stoma Quality of Life Questionnaire (Stoma-QOL)			Exercise of Self-care Agency scale (ESCA)	Incidence proportion of any complication (peristomal irritant dermatitis, peristomal hernia, stoma prolapse, stoma stenosis, urine dermatitis, Stoma bleeding, candida infection)			Resilience: Connor Davidson Resilience scale	Baseline (admission), hospital discharge, 3 and 6 months after discharge.
3	Andrews GR, Sharma A. 2018				Structured Knowledge questionnaire				Coping checklist for patients, Attitude scale, Physical problems checklist	Baseline (within 6 months after surgery), 30 days, 60 days and 90 days after interventio n.
4	Cengiz B, et al. 2020.	Stoma Quality of Life Scale (SQOLS)				Ostomy Complication Severity Index (OCSI)	Ostomy Adjustment Inventory (OAI-23)		Hospital readmissions and cost	Baseline (first home visit after surgery) and 6 months.
5	Cheung YL, et al. 2003.	Quality of Life for colostomy instrument (QOL-colostomy) and World Health Organization Quality of Life-Brief Version (WHOQOL-BREF)	State Trait Anxiety Inventory (STAI)							Baseline (within 1 week after surgery), week 5, and week 10.
6	Duluklu B, et al. 2019	Stoma Quality of Life Scale (SQOLS)					Ostomy Adjustment Inventory (OAI-23)		Presence of odor	Baseline (at least 3 months after surgery) and 1 month after intervention.
7	Huang L, et al. 2018.	European Organization for Research and Treatment of Cancer Quality of Life Questionnaire-Core 30 (EORTC QLQ-C30 V3.0)			Exercise of Self-care Agency scale (ESCA)	Incidence proportion of each type of complication (stomal bleeding, stenosis, edema, ’abscission’; peristomal allergic contact dermatitis)		3-point satisfaction scale (treatment satisfaction)		Baseline (1 day before surgery) and at 4.5 months after surgery.
8	Huang Q, et al. 2021.	Medical Outcomes Study 36-item Short Form Health Survey (SF-36)	Self-rating Anxiety Scale (SAS)	Self-rating Depression Scale (SDS)	Exercise of Self-care Agency scale (ESCA) and General Self-Efficacy Scale (GSES)	Incidence proportion of each type of complication (colostomy infection, stricture, retraction; contact dermatitis)				Pretreatment and at 6 months post-treatment (post discharge)
9	Jiao H, et al. 2020.	Gastrointesti nal Quality of Life Index (GIQLI)	Self-rating Anxiety Scale (SAS)	Self-rating Depression Scale (SDS)	Self-care ability rating scale (no further specified)	Incidence proportion of each type of complication (stoma hemorrhage, ischemia, necrosis, mucocutane ous separation)		3-point satisfaction scale	Comfort: Kolcaba’s General Comfort Questionnaire (GCQ); Happiness: Memorial University of Newfoundland Scale of Happiness (MUNSH)	Baseline (on admission) and after nursing interventio ns (after discharge, but timing is unclear)
10	Jin Y, et al. 2021				Custom Self-care ability checklist for colostomy patients (25 items, self-care knowledge, self-care skills, and self-care attitude)					1 week and 1 month after surgery
11	Li L, et al. 2021.		Self-rating Anxiety Scale (SAS)	Self-rating Depression Scale (SDS)	Custom questionnaire (0–100) Including items on stoma care, scare to stick to the right diet, medication and disease knowledge.			Custom Questionna ire (0–100 score converted to 3 categories of satisfaction)	Surgical indicators (blood loss, ambulation time, GI recovery time, LOS); Nutritional Status (ALB, TFR, PAB); Postoperative recovery.	Baseline (on admission) and 1 month after surgery.
12	Lim SH, et al. 2019	European Organization for Research and Treatment of Cancer Quality of Life Questionnaire Colorectal Cancer 29-item questionnaire (EORTC QLQ-CR29)	Hospital Anxiety and Depression Scales (HADS)	Hospital Anxiety and Depression Scales (HADS)	Stoma Self-Efficacy Scale (Bekkers et al) and Number of days to stoma care proficiency				Acceptance of Chronic Health Conditions Scale (ACHC) Hospital length of stay	Baseline (at enrollment after scheduling for surgery), day of discharge, 4 weeks and 4 months after discharge.
13	Liu H, et al. 2019.	European Organization for Research and Treatment of Cancer Quality of Life Questionnaire-Core 30 (EORTC QLQ-C30 V3.0)				Incidence proportion of each type of complication (Stoma stenosis, prolapse, mucosal separation, retraction; parastomal hernia; skin complications)				Baseline (at discharge), 1 month and 3 months after surgery.
14	Liu Y, Ni L. 2021.	World Health Organization Quality of Life-Brief Version (WHOQOL-BREF)	Hamilton Anxiety Rating Scale (HAM-A)	Hamilton Depression Rating Scale (HAM-D)		Incidence proportion of each type of complication (Parastomal hernia; dermatitis; stomal prolapse, stenosis, retraction; intestinal obstruction)			Defecation regularity	Baseline (during admission) and after interventio n (after surgery, timing is unclear)
15	Lumdubwong A, et al. 2014.	Functional Assessment of Cancer Therapy Colorectal Cancer (FACT-C)				Number of events per group (stoma damage, infection, prolapse, stenosis)			Stress: Perceived Stress Scale (PSS); Coping: The Ways of Coping Questionnai re (WOCQ)	Unclear, at least 6 months after surgery.
16	Wang S, et al. 2021.	Medical Outcomes Study 36-item Short Form Health Survey (SF-36)	Self-rating Anxiety Scale (SAS) and Hamilton Anxiety Rating Scale (HAM-A)	Self-rating Depression Scale (SDS) and Hamilton Depression Rating Scale (HAM-D)					Defecation discomfort and regularity; Nurses’ Observation Scale for Inpatient Evaluation (NOSIE); Sleep Quality: Pittsburgh Sleep Quality Index (PSQI); Employment and empowerme nt levels	Baseline (before surgery) and after interventio n (after surgery, timing is unclear).
17	Xia L. 2020.	Stoma Quality of Life Questionnaire (Stoma-QOL)	State-Trait Anxiety Inventory (STAI)		Stoma Self-Efficacy Scale (Bekkers et al)	Incidence proportion of each type of complication (stomal stenosis, prolapse, retraction; parastomal hernia; skin issues)		5-point satisfaction scale		Baseline (before surgery) and 3 months after surgery.
18	Xu S, et al. 2018.	European Organization for Research and Treatment of Cancer Quality of Life Questionnaire-Core 30 (EORTC QLQ-C30 V3.0)			Stoma Self-Efficacy Scale (Bekkers et al)					10 days, 1 month and 3 months after surgery.
19	Xu W, et al. 2020.	Custom Quality of Life scale	Self-rating Anxiety Scale (SAS)	Self-rating Depression Scale (SDS)	Customized Self-care questionnaire (4dimensions, 50 points each. Self-consciousness, Self-responsibility, Self-care skills, Health knowledge level)	Incidence proportion of any complication (stoma mucosal bleeding, ischemia; mucocutane ous separation; anastomotic stricture)		Custom Questionna ire (18 items with 4-point satisfaction scale)		Baseline (at discharge) and 1 month after surgery.
20	Yu S, et al. 2021.	European Organization for Research and Treatment of Cancer Quality of Life Questionnaire-Core 30 (EORTC QLQ-C30 V3.0)	Self-rating Anxiety Scale (SAS)	Self-rating Depression Scale (SDS)	Self-care ability rating (10 items scored 0–5, Max 50) Higher score means higher self-care ability. <10 severe dependence, 11–20 as moderate, 21–49 as mild, and a total score of 50 as independent	Incidence proportion of each type of complication (fecal dermatitis; stomal prolapse, retraction, stenosis; hernia; surgical incision infection)		3-point satisfaction scale	Recovery of GI function (first time of exhaust, reinitiation of food intake, recovery of bowel sounds). Pain 3 days after surgery: Visual Analogue Scale (VAS)	Baseline (one day before surgery), Pain PO day 3, rest at discharge.
21	Krouse, et al. 2024	City of Hope QOL-Ostomy questionnaire	Hospital Anxiety and Depression Scale	Hospital Anxiety and Depression Scale	Self-efficacy for ostomy patients and Ostomy Knowledge				Patient activation measure	Baseline, postsession, and 6-month follow-up
