# Supplementary material for: Interventions Targeting Quality of Life for Colorectal Cancer Patients with Fecal Ostomy: A Systematic Review
Source: J Gastrointest Surg. Author manuscript; Available in PMC 2026 Jun 18. (PMC13274806; doi:10.1016/j.gassur.2026.102454)
Supplement: 1 — Appendix 1. Search terms used for systematic review query [file NIHMS2176818-supplement-1.docx]

Appendix 1. Search terms used for systematic review query

Medline via Ovid

| **#** | **Query** |
| --- | --- |
| 1 | ("quality of life" or HRQOL or stress or distress or coping or copes or cope).ti,ab. |
| 2 | Quality of life/ or "Stress, Psychological"/ or "Psychological Distress"/ or "Adaptation, Psychological"/ or "Emotional Adjustment"/ |
| 3 | 1 or 2 |
| 4 | (Stoma or stomas or ostom* or ileostom* or colostom* or enterstom* or cecostom* or duodenostom* or jejunostom*).ti,ab. |
| 5 | Surgical stomas/ or ostomy/ or exp Enterostomy/ |
| 6 | 4 or 5 |
| 7 | 3 and 6 |
| 8 | exp "Colorectal Neoplasms"/ |
| 9 | ((colon or colorectal or colonic or colitis or sigmoid or rectal or rectum or rectosigmoid or adenomat*) adj2 (cancer* or neoplas* or malignan* or oncolog* or tumor* or tumour* or adenocarcinoma*)).ti,ab. |
| 10 | 8 or 9 |
| 11 | 7 and 10 |

Cochrane Central Register of Controlled Trials via Ovid

| **#** | **Query** |
| --- | --- |
| 1 | ("quality of life" or HRQOL or stress or distress or coping or copes or cope).ti,ab. |
| 2 | Quality of life/ or "Stress, Psychological"/ or "Psychological Distress"/ or "Adaptation, Psychological"/ or "Emotional Adjustment"/ |
| 3 | 1 or 2 |
| 4 | (Stoma or stomas or ostom* or ileostom* or colostom* or enterstom* or cecostom* or duodenostom* or jejunostom*).ti,ab. |
| 5 | Surgical stomas/ or ostomy/ or exp Enterostomy/ |
| 6 | 4 or 5 |
| 7 | 3 and 6 |
| 8 | exp "Colorectal Neoplasms"/ |
| 9 | ((colon or colorectal or colonic or colitis or sigmoid or rectal or rectum or rectosigmoid or adenomat*) adj2 (cancer* or neoplas* or malignan* or oncolog* or tumor* or tumour* or adenocarcinoma*)).ti,ab. |
| 10 | 8 or 9 |
| 11 | 7 and 10 |

PsycInfo via Ovid

| **#** | **Query** |
| --- | --- |
| 1 | ("quality of life" or HRQOL or stress or distress or coping or copes or cope).ti,ab. |
| 2 | (Stoma or stomas or ostom* or ileostom* or colostom* or enterstom* or cecostom* or duodenostom* or jejunostom*).ti,ab. |
| 3 | ((colon or colorectal or colonic or colitis or sigmoid or rectal or rectum or rectosigmoid or adenomat*) adj2 (cancer* or neoplas* or malignan* or oncolog* or tumor* or tumour* or adenocarcinoma*)).ti,ab. |
| 4 | "health related quality of life"/ or exp "quality of life"/ or "quality of life measures"/ |
| 5 | exp coping behavior/ |
| 6 | exp stress/ |
| 7 | exp "Stress and Coping Measures"/ |
| 8 | colostomy/ |
| 9 | 1 or 4 or 5 or 6 or 7 |
| 10 | 2 or 8 |
| 11 | 3 and 9 and 10 |

CINAHL via EBSCO

| **#** | **Query** |
| --- | --- |
| S1 | TI ( ("quality of life" or HRQOL or stress or distress or coping or copes or cope) ) OR AB ( ("quality of life" or HRQOL or stress or distress or coping or copes or cope) ) |
| S2 | (MH "Quality of Life+") OR (MH "Psychological Well-Being") OR (MH "Stress+") OR (MH "Coping+") |
| S3 | S1 OR S2 |
| S4 | TI ( (Stoma or stomas or ostom* or ileostom* or colostom* or enterstom* or cecostom* or duodenostom* or jejunostom*) ) OR AB ( (Stoma or stomas or ostom* or ileostom* or colostom* or enterstom* or cecostom* or duodenostom* or jejunostom*)) |
| S5 | (MH "Ostomy") OR (MH "Surgical Stoma") OR (MH "Enterostomy+") |
| S6 | S4 OR S5 |
| S7 | (MH "Colorectal Neoplasms+") |
| S8 | TI ( (colon or colorectal or colonic or colitis or sigmoid or rectal or rectum or rectosigmoid or adenomat*) N2 (cancer* or neoplas* or malignan* or oncolog* or tumor* or tumour* or adenocarcinoma*) ) OR AB ( (colon or colorectal or colonic or colitis or sigmoid or rectal or rectum or rectosigmoid or adenomat*) N2 (cancer* or neoplas* or malignan* or oncolog* or tumor* or tumour* or adenocarcinoma*) ) |
| S9 | S7 OR S8 |
| S10 | S3 AND S6 AND S9 |

Embase

| **#** | **Query** |
| --- | --- |
| #1 | 'quality of life'/exp OR 'physiological stress'/exp OR 'coping behavior'/exp |
| #2 | 'quality of life':ab,ti OR hrqol:ab,ti OR stress:ab,ti OR distress:ab,ti OR coping:ab,ti OR copes:ab,ti OR cope:ab,ti |
| #3 | #1 OR #2 |
| #4 | stoma:ab,ti OR stomas:ab,ti OR ostom*:ab,ti OR ileostom*:ab,ti OR colostom*:ab,ti OR enterstom*:ab,ti OR cecostom*:ab,ti OR duodenostom*:ab,ti OR jejunostom*:ab,ti |
| #5 | 'stoma'/de OR 'colon stoma'/exp OR 'ostomy'/de OR 'enterostomy'/exp |
| #6 | #4 OR #5 |
| #7 | 'colorectal cancer'/exp |
| #8 | ((colon OR colorectal OR colonic OR colitis OR sigmoid OR rectal OR rectum OR rectosigmoid OR adenomat*) NEAR/2 (cancer* OR neoplas* OR malignan* OR oncolog* OR tumor* OR tumour* OR adenocarcinoma*)):ab,ti |
| #9 | #7 OR #8 |
| #10 | #3 AND #6 AND #9 |

Web of Science

| **#** | **Query** |
| --- | --- |
| #1 | TS=(("quality of life" or HRQOL or stress or distress or coping or copes or cope)) |
| #2 | TS=(Stoma or stomas or ostom* or ileostom* or colostom* or enterstom* or cecostom* or duodenostom* or jejunostom*) |
| #3 | TS=(((colon or colorectal or colonic or colitis or sigmoid or rectal or rectum or rectosigmoid or adenomat*) NEAR/2 (cancer* or neoplas* or malignan* or oncolog* or tumor* or tumour* or adenocarcinoma*))) |
| #4 | #1 AND #2 AND #3 |
