# Supplementary material for: Interventions Targeting Quality of Life for Colorectal Cancer Patients with Fecal Ostomy: A Systematic Review
Source: J Gastrointest Surg. Author manuscript; Available in PMC 2026 Jun 18. (PMC13274806; doi:10.1016/j.gassur.2026.102454)
Supplement: 2 — Appendix 2. Description of Multidimensional Quality of Life Scales [file NIHMS2176818-supplement-2.docx]

Appendix 2. Description of Multidimensional Quality of Life Scales

| Name | Abbreviation | Description | Scores | Reference |
| --- | --- | --- | --- | --- |
| European Organization for Research and Treatment of Cancer Quality of Life Questionnaire-Core 30 Version 3.0 | EORTC QLQ-C30 V.30 | 30-item instrument designed to measure quality of life in all cancer patients. It includes five functional scales: physical functioning (5 items), role functioning (2 items), cognitive functioning (2 items), emotional functioning (4 items), and social functioning (2 items); three symptom scales: fatigue (3 items), pain (2 items), and nausea and vomiting (2 items); a global health status/QOL scale (2 items); and six single items (dyspnea, appetite loss, sleep disturbance, constipation, diarrhea, and financial impact). | 0-100 | Scott, N. W., Fayers, P., Aaronson, N. K., Bottomley, A. et al. & EORTC Quality of Life Group (2008). EORTC QLQ-C30 Reference Values Manual. (2nd ed.) EORTC Quality of Life Group. http://groups.eortc.be/qol/downloads/reference_values_manual2008.pdf |
| Stoma Quality of Life | Stoma-QOL | 20 items questionnaire to evaluate quality of life in ostomy patients. Four domains – sleep, sexual activity, relations to family and close friends, and social relations to other than family and close friends. | 0-100 | Prieto L, Thorsen H, Juul K. Development and validation of a quality-of-life questionnaire for patients with colostomy or ileostomy. Health Qual Life Outcomes. 2005;3:62. |
| Stoma Quality of Life Scale | SQOLS | 21-item questionnaire to evaluate quality of life in ostomy patients. Three domains: Work/Social Function (6 items), Sexuality/Body Image (5 items), and Stoma Function (6 items). In addition, one item (scored separately) measures financial impact, one measures skin irritation, and two measure overall satisfaction. | 0-100 | Baxter NN, Novotny PJ, Jacobson T et al. (2006) A stoma quality of life scale. Dis Colon Rectum 49:205–212 |
| World Health Organization Quality of Life-Brief Version | WHOQOL-BREF | 26-item questionnaire designed to measure quality of life in various health conditions and healthy individuals. Four domains: physical health (7 items), psychological health (6 items), social relationships (3 items), and environment (8 items). | 0-100 | Development of the World Health Organization WHOQOL-BREF quality of life assessment. The WHOQOL Group. Psychological medicine vol. 28,3 (1998): 551-8. |
| Medical Outcomes Study 36-item Short Form Health Survey | SF-36 | 36-item questionnaire used to assess quality of life in various health conditions and healthy individuals. It evaluates eight domains: Physical Functioning (10 items), Role Limitations Due to Physical Health Problems (4 items), Bodily Pain (2 items), General Health Perceptions (5 items), Vitality (Energy/Fatigue) (4 items), Social Functioning (2 items), Role Limitations Due to Emotional Problems (3 items), and Mental Health (Psychological Distress and Well-Being) (5 items) | 0-100 | Rand Corporation (2019). 36-Item short form survey instrument (SF-36). Rand.org. https://www.rand.org/health-care/surveys_tools/mos/36-item-short-form/survey-instrument.html |
| Gastrointestinal Quality of Life Index | GIQLI | 36-item questionnaire in patients with gastrointestinal disorders. 5 subscales: gastrointestinal symptoms (19 items), physical wellbeing (7 items), emotional wellbeing (5 items), social functioning and medication side-effects (1 item) | 0-144 | Eypasch, E., Williams, J. I., Wood-Dauphinee, S. et al. (1995). Gastrointestinal Quality of Life Index: Development, validation, and application of a new instrument. British Journal of Surgery, 82(2), 216-222. |
| Quality of Life for colostomy instrument | QOL-Colostomy | 23-item questionnaire specifically designed for colostomy patients. Each item uses a 10 cm linear visual analogue scale, measuring seven domains: physical and psychological well-being, body image (colostomy) and social concerns, diagnosis/treatment response (surgical), nutritional response, and overall QOL | 0-230 | Padilla GV, Frank-Stromborg M. 1997. Single instruments for measuring quality of life. In Instruments for Clinical Health-care Research (2nd edn), Frank-Stromborg M, Olsen SJ (eds). Jones & Barlett Publishers: Boston; 114–134. |
| European Organization for Research and Treatment of Cancer Quality of Life Questionnaire Colorectal Cancer 29-item questionnaire | EORTC QLQ-CR29 | 29-item questionnaire designed to measure health-related quality of life in colorectal cancer patients. It complements the EORTC QLQ-C30 questionnaire. Includes four functional scales: body image (1 item), anxiety (1 item), weight (1 item), and sexual interest (2 items: 1 for men and 1 for women). And nineteen symptom items covering gastrointestinal symptoms, urinary symptoms, sexual problems, and chemotherapy side effects. | 0-100 | Gujral S, Conroy T, Fleissner C et al. Assessing quality of life in patients with colorectal cancer: An update of the EORTC quality of life questionnaire. Eur J Cancer 2007;29:276–81. |
| The Functional Assessment of Cancer Therapy (General) | FACT-G | 27-item questionnaire designed to measure four domains of HRQOL in cancer patients: Physical, social, emotional, and functional well-being. (0-4 Likert scale) | 0-108 | Cella D.F., Tulsky D.S., Gray G. et al. The Functional Assessment of Cancer Therapy (FACT) Scale: Development and validation of the general measure. *Journal of Clinical Oncology*1993; 11(3): 570-579. |
| City of Hope Quality of Life-Ostomy questionnaire | COH-QOL-O | 43-item revised questionnaire designed to measure four domains of HR QOL in ostomy patients: physical, psychological, social, and spiritual well-being. (0-10 scale) | 0-10 | Grant M, Ferrell B, Dean G, Uman G, Chu D, Krouse R. Revision and psychometric testing of the city of hope quality of life-ostomy questionnaire. *Qual Life Res*. 2004; **13**(8): 1445-1457 |
