# Supplementary material for: Interventions Targeting Quality of Life for Colorectal Cancer Patients with Fecal Ostomy: A Systematic Review
Source: J Gastrointest Surg. Author manuscript; Available in PMC 2026 Jun 18. (PMC13274806; doi:10.1016/j.gassur.2026.102454)
Supplement: 3 — Appendix 3. Bias Assessment [file NIHMS2176818-supplement-3.docx]

Appendix 3. Bias Assessment

| **Domain 1.** **Randomization Process** | | | | | | | | | | | | | | | | | | | | | |
| --- | --- | --- | --- | --- | --- | --- | --- | --- | --- | --- | --- | --- | --- | --- | --- | --- | --- | --- | --- | --- | --- |
| **Study Number** | **1** | **2** | **3** | **4** | **5** | **6** | **7** | **8** | **9** | **10** | **11** | **12** | **13** | **14** | **15** | **16** | **17** | **18** | **19** | **20** | **21** |
| 1.1 Was the allocation sequence random? | Y | Y | N | N | Y | N | Y | N | Y | N | NI | Y | Y | Y | N | Y | Y | Y | N | Y | Y |
| 1.2 Was the allocation sequence concealed until participants were enrolled and assigned to interventions? | Y | PY | N | NI | PY | NI | PY | NI | PY | NI | NI | Y | PY | PY | NI | PY | Y | Y | NI | PY | Y |
| 1.3 Did baseline differences between intervention groups suggest a problem with the randomization process? | N | N | PY | PY | PY | N | N | N | N | N | N | PY | N | N | N | N | N | N | N | N | N |
| Risk-of-bias judgement | Low | Low | High | High | SC | SC | Low | SC | Low | SC | SC | SC | Low | Low | SC | Low | Low | Low | SC | Low | Low |
| **Domain 2. Risk of bias due to deviations from the intended interventions (effect of adhering to intervention)** | | | | | | | | | | | | | | | | | | | | |  |
| **Study Number** | **1** | **2** | **3** | **4** | **5** | **6** | **7** | **8** | **9** | **10** | **11** | **12** | **13** | **14** | **15** | **16** | **17** | **18** | **19** | **20** | **21** |
| 2.1. Were participants aware of their assigned intervention during the trial? | PY | PY | PY | PY | PY | Y | PY | PY | PY | PN | PY | N | PY | Y | N | Y | N | Y | PN | PY | PY |
| 2.2. Were carers and people delivering the interventions aware of participants' assigned intervention during the trial? | PY | PY | PY | PY | PY | Y | PY | PY | Y | Y | PY | PN | PY | Y | N | Y | N | Y | PN | PY | PY |
| 2.3. [If applicable:] If Y/PY/NI to 2.1 or 2.2: Deviations that arose because of the trial context? | N | N | N | N | N | N | N | N | N | N | N |  | N | N |  | N |  | N |  | N | N |
| 2.4. [If applicable:] Were there failures in implementing the intervention that could have affected the outcome? |  |  |  |  |  |  |  |  |  |  |  |  |  |  |  |  |  |  |  |  | PY |
| 2.5. [If applicable:] Was there non-adherence to the assigned intervention regimen that could have affected participants’ outcomes? |  |  |  |  |  |  |  |  |  |  |  |  |  |  |  |  |  |  |  |  |  |
| 2.6. If N/PN/NI to 2.3, or Y/PY/NI to 2.4 or 2.5: Was an appropriate analysis used to estimate the effect of adhering to the intervention? |  |  |  |  |  |  |  |  |  |  |  |  |  |  |  |  |  |  |  |  |  |
| Risk-of-bias judgement | Low | Low | Low | Low | Low | Low | Low | Low | Low | Low | Low | Low | Low | Low | Low | Low | Low | Low | Low | Low | Low |
| **Domain 3. Missing outcome data** | | | | | | | | | | | | | | | | | | | | |  |
| **Study Number** | **1** | **2** | **3** | **4** | **5** | **6** | **7** | **8** | **9** | **10** | **11** | **12** | **13** | **14** | **15** | **16** | **17** | **18** | **19** | **20** | **21** |
| 3.1 Were data for this outcome available for all, or nearly all, participants randomized? | Y | Y | Y | N | Y | Y | Y | Y | Y | Y | Y | Y | Y | Y | Y | Y | Y | Y | Y | Y | Y |
| 3.2 If N/PN/NI to 3.1: Is there evidence that the result was not biased by missing outcome data? |  |  |  | NI |  |  |  |  |  |  |  |  |  |  |  |  |  |  |  |  |  |
| 3.3 If N/PN to 3.2: Could missingness in the outcome depend on its true value? |  |  |  | PN |  |  |  |  |  |  |  |  |  |  |  |  |  |  |  |  |  |
| 3.4 If Y/PY/NI to 3.3: Is it likely that missingness in the outcome depended on its true value? |  |  |  |  |  |  |  |  |  |  |  |  |  |  |  |  |  |  |  |  |  |
| Risk-of-bias judgement | Low | Low | Low | Low | Low | Low | Low | Low | Low | Low | Low | Low | Low | Low | Low | Low | Low | Low | Low | Low | Low |
| **Domain 4.** **Risk of bias in measurement of the outcome** | | | | | | | | | | | | | | | | | | | | |  |
| **Study Number** | **1** | **2** | **3** | **4** | **5** | **6** | **7** | **8** | **9** | **10** | **11** | **12** | **13** | **14** | **15** | **16** | **17** | **18** | **19** | **20** | **21** |
| 4.1 Was the method of measuring the outcome inappropriate? | N | N | N | N | N | N | N | N | N | N | N | N | N | N | N | N | N | N | N | N | N |
| 4.2 Could measurement or ascertainment of the outcome have differed between intervention groups? | N | N | N | N | N | N | N | N | N | N | N | N | N | N | PN | N | N | N | N | N | N |
| 4.3 If N/PN/NI to 4.1 and 4.2: Were outcome assessors aware of the intervention received by study participants? | Y | Y | Y | Y | Y | N | Y | Y | Y | PY | PY | N | PY | PY | PN | PY | N | PY | PY | Y | PY |
| 4.4 If Y/PY/NI to 4.3: Could assessment of the outcome have been influenced by knowledge of intervention received? | N | N | N | N | N |  | N | N | N |  | N |  | N | N | N | N |  | N | N | N | N |
| 4.5 If Y/PY/NI to 4.4: Is it likely that assessment of the outcome was influenced by knowledge of intervention received? |  |  |  |  |  |  |  |  |  |  |  |  |  |  |  |  |  |  |  |  |  |
| Risk-of-bias judgement | Low | Low | Low | Low | Low | Low | Low | Low | Low | Low | Low | Low | Low | Low | Low | Low | Low | Low | Low | Low | Low |
| **Domain 5. Risk of bias in selection of the reported result** | | | | | | | | | | | | | | | | | | | | |  |
| **Study Number** | **1** | **2** | **3** | **4** | **5** | **6** | **7** | **8** | **9** | **10** | **11** | **12** | **13** | **14** | **15** | **16** | **17** | **18** | **19** | **20** | **21** |
| 5.1 Were the data that produced this result analyzed in accordance with a pre-specified analysis plan that was finalized before unblinded outcome data were available for analysis? | PY | PY | PY | PY | Y | Y | Y | Y | Y | Y | Y | Y | Y | Y | Y | Y | Y | Y | Y | PY | Y |
| Is the numerical result being assessed likely to have been selected, on the basis of the results, from... | | | | | | | | | | | | | | | | | | | | |  |
| 5.2. ... multiple eligible outcome measurements (e.g. scales, definitions, time points) within the outcome domain? | N | N | N | N | Y | N | N | N | N | N | N | N | N | N | N | Y | N | N | N | N | Y |
| 5.3 ... multiple eligible analyses of the data? | N | N | N | N | Y | N | N | N | N | N | N | N | N | N | N | N | N | N | N | N | N |
| Risk-of-bias judgement | Low | Low | Low | Low | High | Low | Low | Low | Low | Low | Low | Low | Low | Low | Low | Low | Low | Low | Low | Low | Low |
| **Study Number** | **1** | **2** | **3** | **4** | **5** | **6** | **7** | **8** | **9** | **10** | **11** | **12** | **13** | **14** | **15** | **16** | **17** | **18** | **19** | **20** | **21** |
| **Overall risk of bias** | Low | Low | High | High | SC | SC | Low | SC | Low | SC | SC | SC | Low | Low | SC | Low | Low | Low | Low | Low | Low |

*Y: Yes; N: No; PY: probably yes; PN: probably no; NI: no information; SC: some concerns


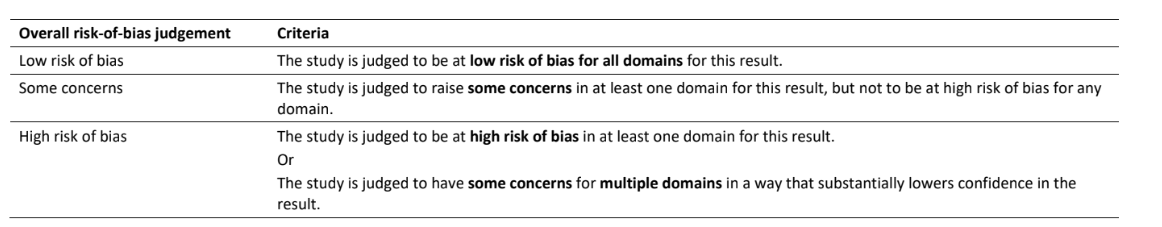


Sterne JAC, Savović J, Page MJ, et al. RoB 2: a revised tool for assessing risk of bias in randomized trials. BMJ. 2019;l4898.
