# Supplementary material for: Interventions Targeting Quality of Life for Colorectal Cancer Patients with Fecal Ostomy: A Systematic Review
Source: J Gastrointest Surg. Author manuscript; Available in PMC 2026 Jun 18. (PMC13274806; doi:10.1016/j.gassur.2026.102454)
Supplement: 4 — Appendix 4. Inclusion criteria [file NIHMS2176818-supplement-4.docx]

Table 2. Inclusion criteria

|  | Study | Age (years) | Colorectal diagnosis | Type of surgery | Stoma permanency | Elective/  Emergent | Other Inclusion criteria | Exclusion criteria |
| --- | --- | --- | --- | --- | --- | --- | --- | --- |
| 1 | Su X, et al. 2021 | >18 | Rectal cancer | n-rLAR | Temporary | Elective | At least 18 years old; could read and write; interested in participating in the study; diagnosed with rectal cancer; underwent anterior resection; underwent temporary stomas; expected to survive for more than 1 year; could be contacted via telephone after discharge. | Other critical diseases; unable to take care of themselves; abusing alcohol or drugs; suffered from a psychiatric disease; lived in a nursing home |
| 2 | Zhang X, et al. 2020 | >18 | Colorectal cancer (ICD10 code C18) | Unspecified | Permanent | Elective | Pathologically diagnosed with colorectal cancer (ICD-10 code C18) for the first time and willing to receive a permanent enterostomy; no previous history of any malignant tumor and chronic disease; over 18 years of age; stable postoperative conditions and no serious complications; ability of oral communication; knowledge of the disease condition and willingness to participate in the intervention | Previous neoadjuvant chemotherapy; illiterate; admitted to the ICU after surgery; discharge right after surgery; history of neurological disease or mental illness; participation in other psychosocial support therapy; unable to continue in study due to physical discomfort; use of psychotropic meds during intervention. |
| 3 | Andrews GR, Sharma A. 2018 | >18 | "Cancer patients" | Unspecified | Unspecified | Unspecified | Adults >18 years; colostomy for <6 months; able to speak and read English or Hindi | Visual and hearing or cognitive impairments |
| 4 | Cengiz B, et al. 2020. | >18 | Colorectal cancer | Unspecified | Temporary and permanent (> 3 months) | Unspecified | Willing to participate in the study; >18 years, diagnosed with colorectal cancer, colostomy or ileostomy created in the last 3 months; residing in Izmir; able to read and write in Turkish. | Previously participated in a home care program; stoma reversal during follow-up period. |
| 5 | Cheung YL, et al. 2003. | Adults | "Undergone stoma for cancer treatment" | Unspecified | Temporary and permanent | Elective | Underwent temporary or permanent stoma surgery for cancer treatment in the selected regional hospitals; adults; either sex; willing to participate. | Uncontrolled heart disease; diabetes mellitus; disorientation; documented psychiatric illness or metastases in CNS; illiterate; hearing difficulty. |
| 6 | Duluklu B, et al. 2019 | > 18 | Colorectal cancer | n-rLAR (n=4, 13.3%) APR (n=26, 86.7%) | Permanent | Unspecified | Opened permanent colostomy >3 months; no mental or communication problem; no complications due to stoma; no chemotherapy during the study; no discomfort from smell of lavender oil; no allergy to lavender oil; residing in city where study was conducted; volunteered to participate | Using any product in the stoma bag to prevent odor; residing in city other than where study was conducted; late complications of ostomy (prolapse, parastomal hernia, stenosis, obstruction, peristomal skin problems, stomal fistula, metabolic complications) |
| 7 | Huang L, et al. 2018. | 18-80 | Colorectal Cancer | APR | Permanent | Unspecified | Could communicate normally and make decisions by themselves; no severe bleeding, infection, or other complications after operation | Serious diseases (uremia, coagulation dysfunction, heart disease); psychiatric disorders; other tumors; disability; not cooperating with medical staff. |
| 8 | Huang Q, et al. 2021. | 20-70 | Rectal cancer | Unspecified | Permanent | Unspecified | No tumor recurrence or metastasis; signed informed consent voluntarily. | Disturbance in communication, comprehension, or reading; underwent radiotherapy, chemotherapy, or immunotherapy; dysfunction of vital organs (heart, liver, kidney, or lung). |
| 9 | Jiao H, et al. 2020. | Unspecified | Colon cancer | Unspecified | Unspecified | Elective | Colostomy patients with confirmed indications for colostomy without distant or systemic metastasis; expected survival time of 1 year at least | Severe infectious disease; neurological dysfunction; cognitive dysfunction; or other vital organ dysfunction; incomplete clinical data; pregnant or lactating women. |
| 10 | Jin Y, et al. 2021 | Unspecified | Rectal cancer | Unspecified | Unspecified | Elective | Aware of disease and normal communication skills; volunteered to participate in the study. | Cognitive impairment or mental abnormality; other malignant tumors or severe heart, brain, liver, kidney, or lung diseases; illiteracy, upper limb disability or poor self-care ability. |
| 11 | Li L, et al. 2021. | Unspecified | Rectal cancer | Radical resection for rectal cancer | Permanent | Elective | Could express their feelings and with normal mental status. | Expected survival <6 months; other diseases that affect the study; communication problems. |
| 12 | Lim SH, et al. 2019 | Unspecified | Rectal cancer (n=41, 80.4%), Sigmoid cancer (n=7, 13.7%) and Rectosigmoid cancer (n=3, 5.9%) | n-rLAR (n=31, 60.8%)  APR (n=13. 25.5%) | Temporary (n=37, 72.5%) and Permanent (n=14, 27.5%) | Elective | Could converse in English or Mandarin Chinese | Complications delayed >5 days before attaining stoma proficiency; communication issues (language, dementia, hearing impairment). |
| 13 | Liu H, et al. 2019. | 18-70 | Colorectal cancer | APR | Permanent | Elective | Able to read and write | Severe cardiovascular, endocrine, or other malignant conditions; emotional or cognitive impairments. |
| 14 | Liu Y, Ni L. 2021. | Unspecified | Colorectal cancer | Unspecified | Unspecified | Unspecified | No language expression disorder; no other organ metastasis in abdominal US or CT; estimated survival time >3 months; signed informed consent. | Stoma complications when discharged; severe heart, liver, lung, kidney and other important organ disorders; other serious somatic diseases; family or personal history of mental disease; cognitive impairment; altered consciousness. |
| 15 | Lumdubwong A, et al. 2014. | >30 | Colorectal cancer | Unspecified | Unspecified (Colostomy for >6 months) | Unspecified | Age 30 years and older; colostomy > 6 months; and orientated to time, place, and person. | Altered consciousness |
| 16 | Wang S, et al. 2021. | Unspecified | Rectal cancer | APR | Permanent | Elective | Either sex; underwent neoadjuvant chemoradiation followed by resection surgery. | Mental disorders or cognitive dysfunction, patients with severe infectious diseases, and patients with other malignant tumors. |
| 17 | Xia L. 2020. | 18-70 | Rectal cancer | APR | Permanent | Elective | Able to read and write and understand the questionnaire; willing to participate in an extra session about colostomy care. | Serious cardiovascular, endocrine, or other serious disease; presence of emotional or cognitive disorders that interfere with the ability to complete the study. |
| 18 | Xu S, et al. 2018. | Unspecified | Colorectal cancer | APR | Permanent | Unspecified | Informed consent and volunteered to participate in the project; able to communicate and fill in the questionnaires alone or under the guidance of the researchers; conscious and had no severe complications during the postoperative recovery period; able to take care of themselves before the operation. | Mental illness; acute or chronic diseases affecting their ability to carry out daily activities |
| 19 | Xu W, et al. 2020. | Unspecified | Rectal cancer | Rectal resection | Permanent | Unspecified | Without serious postoperative complications; good treatment compliance and communication ability. | Presence of heart, liver, kidney or mental disease, or other serious diseases; severe bleeding and/or infection after surgery, coagulation disorders; incomplete data |
| 20 | Yu S, et al. 2021. | Unspecified | Colon cancer (n=15, 50%) Rectal cancer (n=15, 50%) | Radical resection | Unspecified (colostomy for the first time) | Elective | Underwent colostomy surgery for the first time. | Severe cardiopulmonary or other severe organ diseases; altered consciousness; communication disorders; contraindication for surgery. |
| 21 | Krouse, et al. 2024. | >21 years | “cancer survivors” | Unspecified | Temporary or permanent 6+ weeks after surgery | Unspecified | Cancer survivors at least 6 weeks after an operation that led to an ostomy (fecal or urinary) at three tertiary hospitals. | No diagnosis of cancer |

n-rLAR: Non-restorative Lower anterior resection (Hartmann's operation); APR: Abdominoperineal resection (Miles operation); ICU: Intensive Care Unit; CNS: Central Nervous System; US: Ultrasonography; CT: Computed Tomography
